# Complex Management of Brain Metastases in Head and Neck Squamous Cell Carcinoma: A Case Report and Literature Review

**DOI:** 10.7759/cureus.73033

**Published:** 2024-11-05

**Authors:** Giuseppe Corazzelli, Federico Russo, Sergio Corvino, Cristiana Germano, Filippo Dello Iacovo, Valentina Cioffi, Salvatore Di Colandrea, Paola Bonavolontà, Oreste De Divitiis, Raffaele De Falco, Antonio Bocchetti

**Affiliations:** 1 Department of Neurosciences and Reproductive and Odontostomatological Sciences, Neurosurgical Clinic, University of Naples Federico II, Naples, ITA; 2 Department of Public Health, Division of Pathological Anatomy, University of Naples Federico II, Naples, ITA; 3 Department of Neurosurgery, Santa Maria delle Grazie Hospital, Azienda Sanitaria Locale Napoli 2 Nord, Pozzuoli, ITA; 4 Department of Anesthesiology and Intensive Care Medicine, Santa Maria delle Grazie Hospital, Azienda Sanitaria Locale Napoli 2 Nord, Pozzuoli, ITA

**Keywords:** brain metastases, central nervous system metastasis, cyberknife radiosurgery, head and neck squamous cell carcinoma, larynx carcinoma

## Abstract

Head and neck squamous cell carcinoma (HNSCC) can, in rare instances, metastasize to the CNS, which is often associated with a poor prognosis. We present the case of a 65-year-old male with a history of HNSCC who developed two enhancing brain lesions: one in the right parietal region and another in the right insular region. Initially, the patient was managed with CyberKnife radiosurgery targeting both lesions. However, he later experienced neurological symptoms, including confusion, gait instability, and a left Jacksonian march, primarily attributed to the progression of the right parietal lesion. Given this symptomatic presentation, surgical resection of the lesion was performed to confirm the diagnosis and provide symptomatic relief. The surgical approach was selected based on the patient’s clinical progression and imaging findings, with the goal of improving his quality of life. Despite surgical intervention, the patient’s condition deteriorated, and he passed away seven months later. This case illustrates the complexity of treating CNS metastases from HNSCC, highlighting the challenges in surgical decision-making and the role of adjuvant therapies. The rarity of CNS metastases in HNSCC adds to the difficulty of management, underscoring the importance of a multidisciplinary approach. Further investigation is needed to develop standardized treatment protocols for such rare presentations. CNS metastases in HNSCC are uncommon and typically indicate a poor outcome. This case reinforces the need for ongoing research to enhance management strategies and improve patient survival in similar cases.

## Introduction

Head and neck squamous cell carcinoma (HNSCC) encompasses a diverse group of malignancies arising from the mucosal surfaces of the oral cavity, pharynx, and larynx [1]. It is globally recognized as the sixth most common cancer type, accounting for an estimated 600,000 new cases and 350,000 deaths annually [2]. The etiology of HNSCC is multifactorial, with tobacco use and alcohol consumption being the primary risk factors, contributing significantly to the disease burden [3].

Despite advancements in diagnostic and therapeutic strategies, the prognosis for HNSCC remains challenging, especially with the development of distant metastases [4]. While the lungs, liver, and bones are common sites for metastatic spread, CNS involvement is exceedingly rare, occurring in approximately 1% of all HNSCC metastases [5]. Such occurrences are infrequent and are associated with a poor prognosis, with median survival times following the diagnosis of CNS metastases as low as four months [6].

The management of HNSCC with CNS metastases is complex and often individualized due to the scarcity of cases and the absence of standardized treatment protocols [7]. Surgical intervention remains the cornerstone for solitary brain metastases, potentially extending survival [8]. Adjuvant therapies, such as whole brain radiation therapy (WBRT), have traditionally followed surgical resection, despite concerns regarding neurocognitive decline [9]. Recent evidence suggests that stereotactic radiosurgery (SRS) may offer comparable survival benefits with reduced neurotoxicity [10].

This case report details the intricate clinical journey of a patient with high-grade laryngeal carcinoma, exploring the diagnostic challenges and therapeutic decisions encountered in the context of rare brain metastases. It aims to contribute to the limited knowledge of CNS metastases from HNSCC and underscore the need for further research to enhance patient outcomes.

## Case presentation

We present the case of a 65-year-old male patient with a significant history of alcoholism and tobacco use, quantified at 50 pack-years (two packs per day for 25 years). His medical journey with head and neck malignancies began in 2014 with the diagnosis and subsequent asportation of laryngeal carcinoma. Disease progression led to a partial laryngectomy and bilateral neck dissection in 2017, addressing PT3N0M0 squamous cell carcinoma.

During routine follow-up, imaging studies revealed right parietal and insular brain metastases. These were managed noninvasively with CyberKnife radiosurgery in 2022. However, the patient’s condition took a turn for the worse in 2023 when he was admitted to our neurosurgery unit with symptoms of confusion, gait instability, and a left Jacksonian march. A neurological examination revealed left-sided somatic hyposthenia and hyperreflexia.

**Figure 1:**
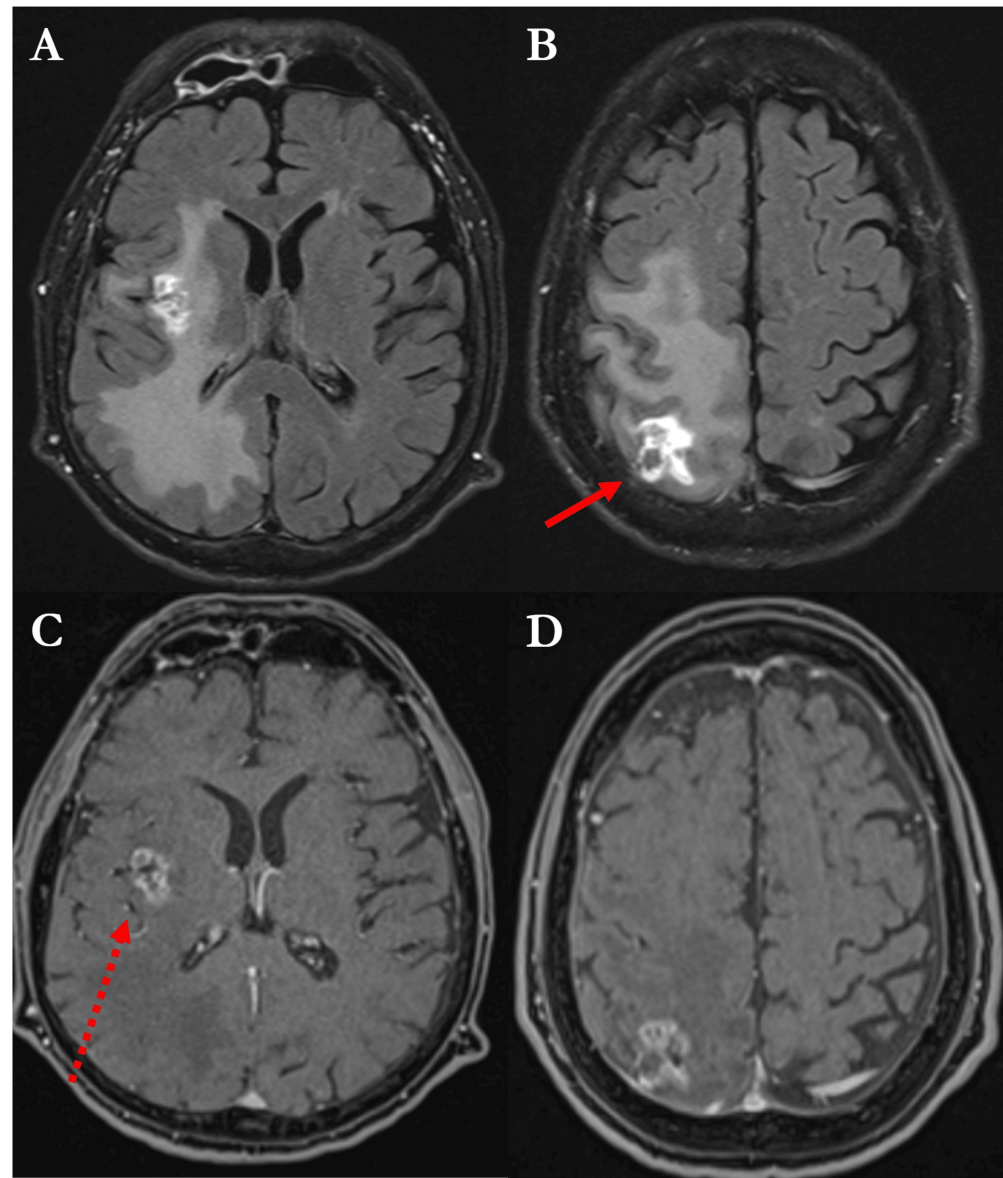
Preoperative contrast-enhanced brain MRI scan (A, B) Axial T2-weighted FLAIR sequences demonstrating a heterogeneously enhancing right parietal lesion characterized by central necrosis and surrounding vasogenic edema (solid arrow). (C, D) Axial T1-weighted contrast-enhanced images depicting the contrast-enhancing right parietal lesion.

Diagnostic imaging, including a brain CT scan followed by MRI, identified heterogeneously enhancing lesions in the right parietal and insular regions, characterized by central necrosis and significant perilesional vasogenic edema (Figure 1).

**Figure 2:**
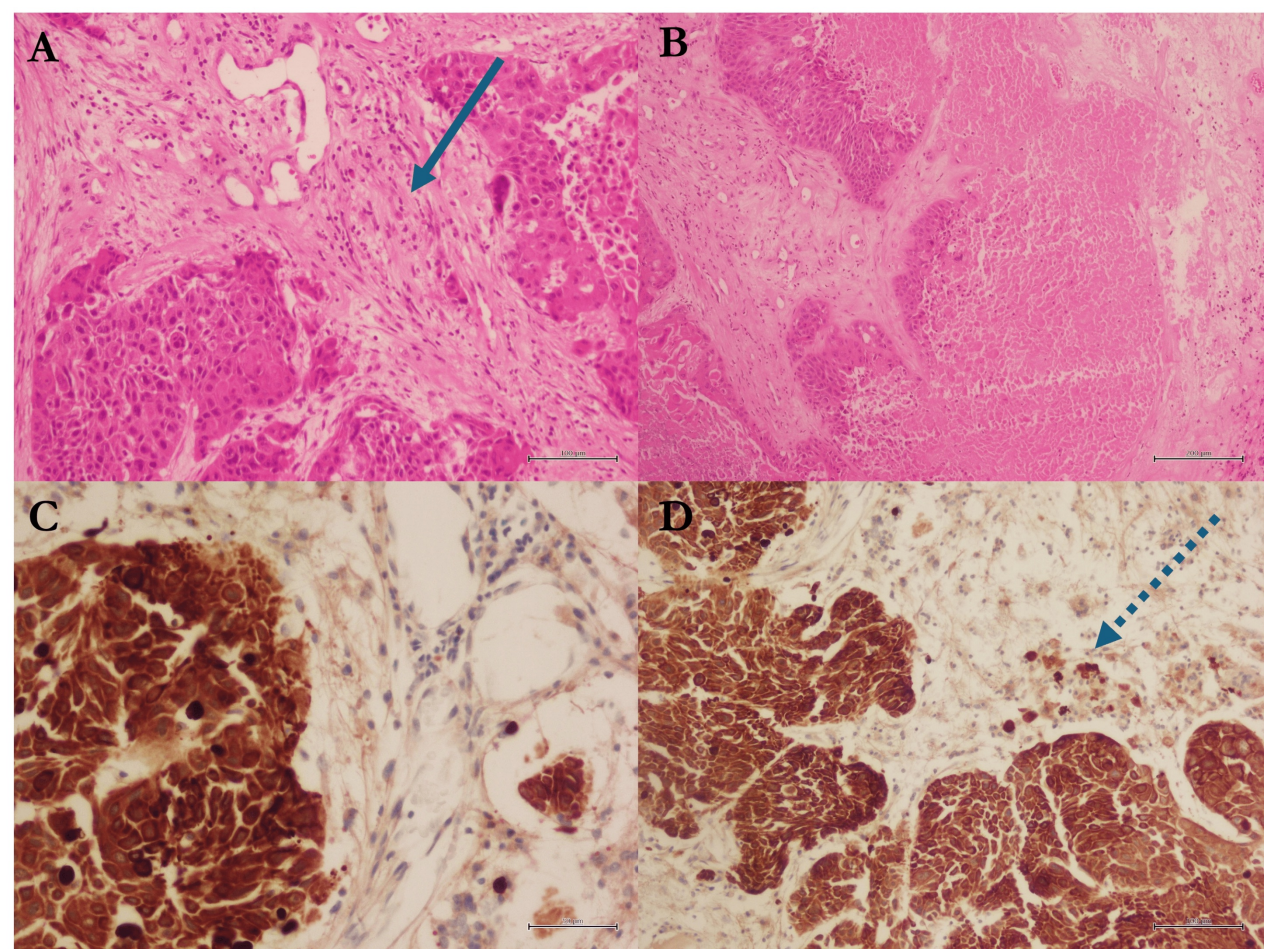
Histopathological examination of the sampled lesion (A, B) H&E-stained sections (A: ×20; B: ×10) demonstrating features of squamous cell carcinoma. The straight arrow in (A) indicates areas of keratin pearl formation, while (B) highlights regions of necrosis. (C, D) CK immunohistochemical staining (C: ×20; D: ×10) showing strong cytoplasmic positivity. The dashed arrow in (D) highlights the contrast between tumor cells and adjacent normal tissue. CK: cytokeratin

A decision was made to perform a craniotomy to remove the parietal lesion while leaving the insular metastasis intact due to its challenging location. The excised tumor was grayish, parenchymatous, and inseparable from the surrounding brain tissue. Pathological analysis confirmed the laryngeal origin of the metastatic lesion, classifying it as high-grade HNSCC (Figure 2).

**Figure 3:**
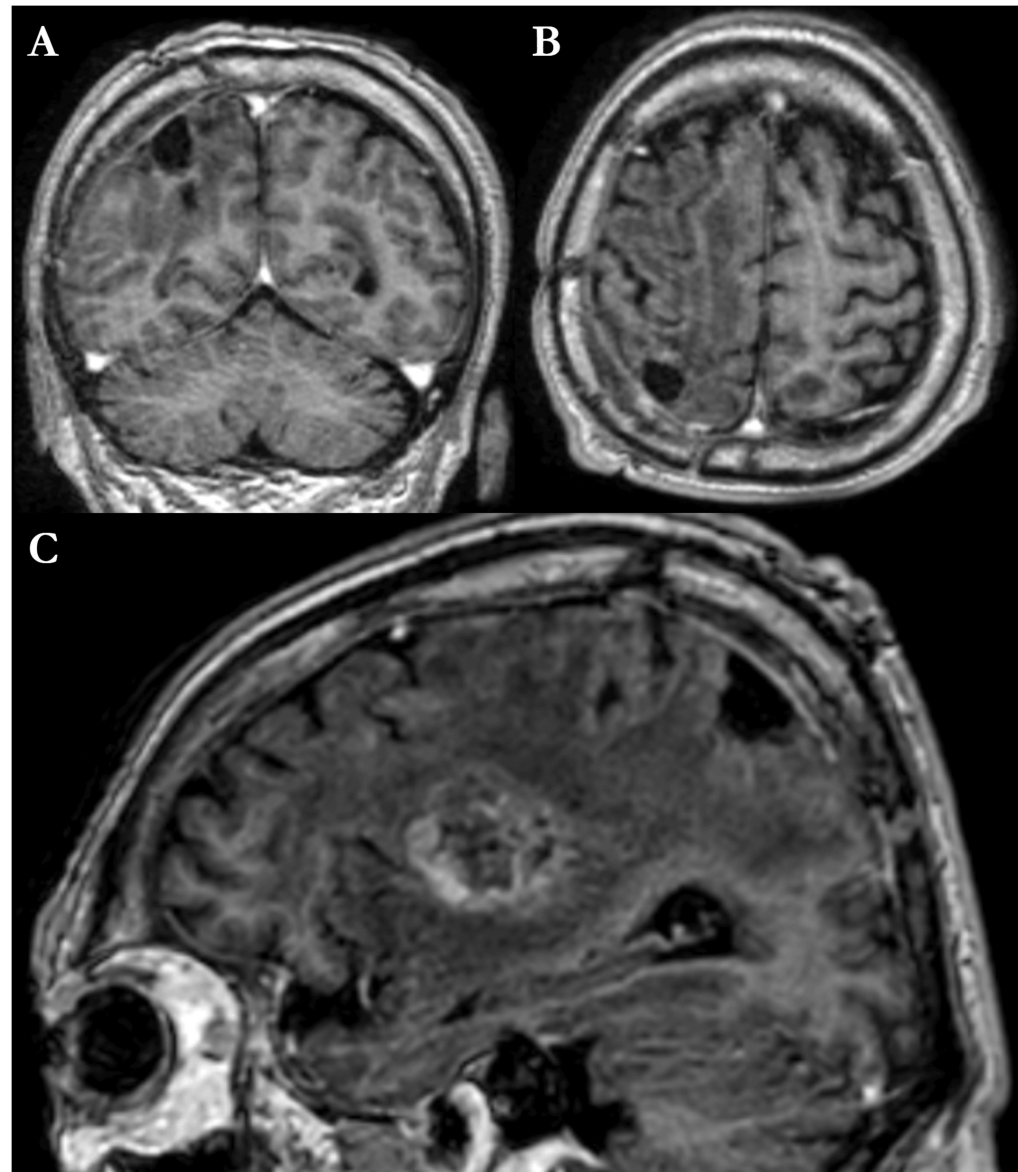
Early postoperative contrast-enhanced brain MRI (A) Frontal T1-weighted contrast-enhanced sequence. (B) Axial T1-weighted contrast-enhanced sequence. (C) Right paramedian sagittal T1-weighted contrast-enhanced sequence. Early postoperative contrast-enhanced brain MRI demonstrated the complete removal of the right parietal lesion, while the right insular lesion exhibited a volumetric increase.

Postoperative MRI scans demonstrated successful resection of the parietal lesion while preserving the insular metastasis (Figure 3).

Unfortunately, the patient’s neurological status declined rapidly following surgery, resulting in significant functional impairment. A comprehensive total-body CT scan revealed no additional metastatic sites. Despite the absence of further lesions, the patient’s deteriorating condition precluded him from receiving adjuvant chemotherapy. Consequently, he underwent WBRT, receiving a total dose of 30 Gy in 10 fractions.

**Figure 4:**
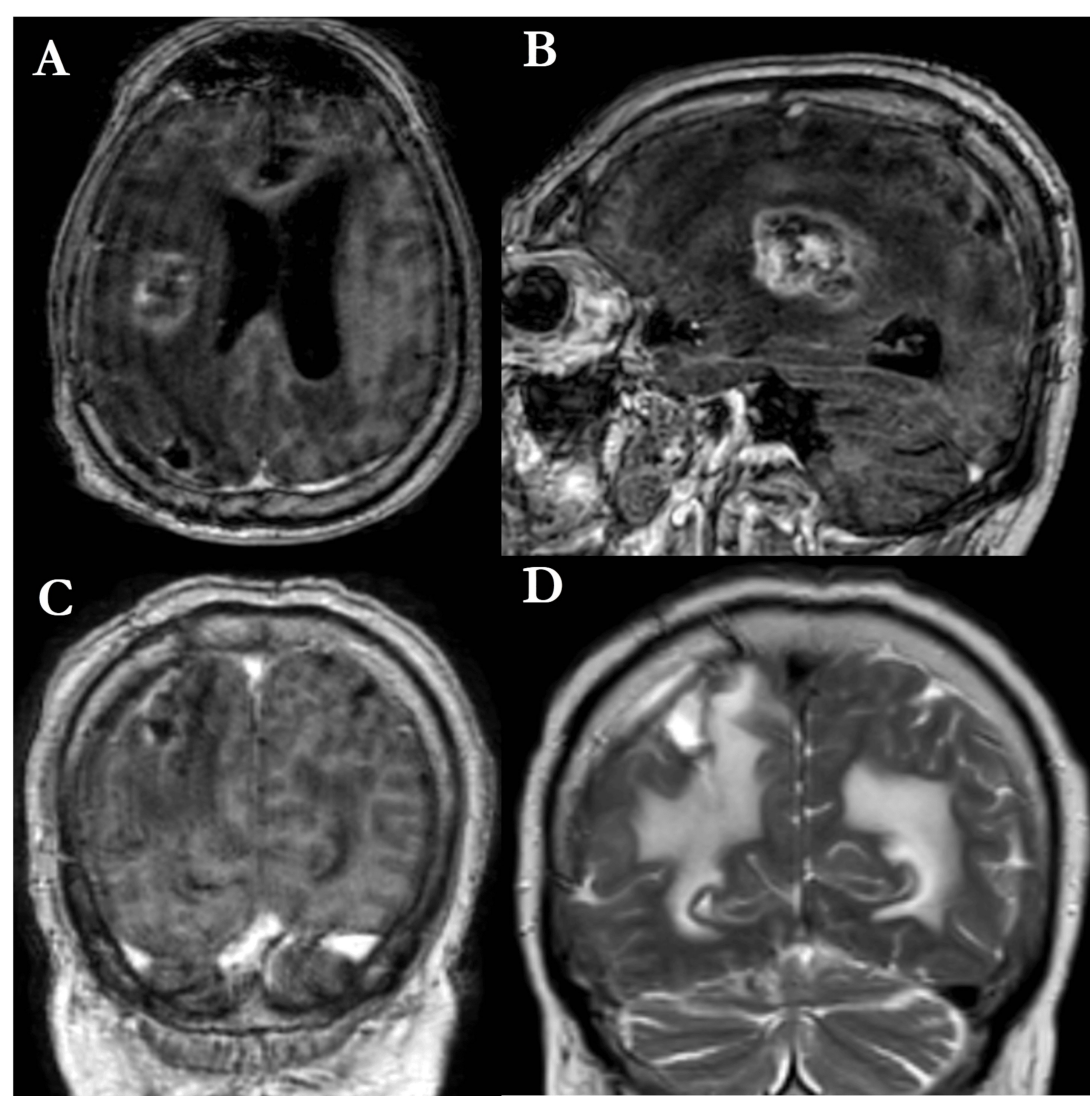
Three months postoperative and post-radiation therapy brain contrast-enhanced MRI (A) Axial T1-weighted contrast-enhanced sequence. (B) Right paramedian sagittal T1-weighted contrast-enhanced sequence. (C) Frontal T1-weighted contrast-enhanced sequence. (D) Frontal T2-weighted FLAIR sequence. The quality of the examination was affected by the patient’s agitated state. The post-radiation therapy brain MRI revealed that the insular lesion had increased in size, and peripheral contrast enhancement in the surgical bed was observed, indicating either radionecrosis or a relapse of the previously removed lesion.

Three months after WBRT, the patient developed generalized seizures. Subsequent imaging indicated growth of the insular lesion and peripheral contrast enhancement at the surgical site, raising concerns of either radionecrosis or recurrence (Figure 4).

Given the patient’s declining neurological state, the medical team opted to cease active treatment and transition to best supportive care. The patient sadly passed away seven months after the surgery, likely due to complications related to the progression of the underlying HNSCC. Presumably, the causes of death included respiratory complications and cachexia.

## Discussion

The case of our 65-year-old patient with high-grade squamous cell carcinoma of the larynx and subsequent CNS metastases offers a valuable opportunity to discuss the complexities involved in managing such rare and intricate conditions [1]. Brain metastases in HNSCC are infrequent, with a reported incidence of only 1% [11]. This rarity presents significant challenges in establishing standardized treatment protocols and contributes to the poor prognosis typically associated with this condition [12-15].

Despite the initial success of CyberKnife radiosurgery in addressing the right parietal and insular metastases, the patient’s neurological status deteriorated following surgical intervention. This outcome raises important questions about the optimal timing and extent of surgical resection, particularly in cases involving multiple brain lesions. The decision to forgo further aggressive treatment in favor of best supportive care was made in light of the patient’s rapidly declining neurological function and the absence of systemic disease.

Recent advancements in the treatment of brain metastases, including SRS techniques like CyberKnife, hold promise for improved outcomes with reduced neurocognitive toxicity compared to traditional WBRT [9,13-20]. However, the effectiveness of SRS in the context of HNSCC metastases to the CNS remains to be fully evaluated, given the limited number of cases and the lack of randomized controlled trials [13-17,21,22].

To contextualize our case within the broader landscape of HNSCC-related CNS metastases, we conducted a literature review spanning from 1980 to 2024. Our review adhered to the CARE guidelines for case reports, ensuring methodological rigor. The final compilation included 42 documented cases, providing a valuable repository of clinical data for further analysis (Table 1).

**Table 1:** Summary of the sampled cases from the literature on HNSCC intracranial metastases CS: cavernous sinus; CT: chemotherapy; EBRT: external-beam radiotherapy; ND: neck dissection; PG: pituitary gland; PL: partial laryngectomy; PP: partial pharyngectomy; TL: total laryngectomy; TP: total pharyngectomy

Author and year	Sex and age	Primary tumor histology	Treatment of primary tumor	Time to metastasis detection (mt)	Signs and symptoms	Site of metastasis	Treatment for CNS metastasis	Mortality (mt)
Ahmad et al. (1984) [18]	M, 70	Supraglottic SCC	EBRT	3	Left blindness and ophthalmoplegia	CS	EBRT	NA
Warwick-Brown and Cheesman (1987) [22]	F, 62	Supraglottic SCC	TL + TP + EBRT	5	Right III palsy, retroocular pain	CS + bilateral temporal-basal	-	2
Bakshi et al. (2009) [12]	M, 64	Glottic SCC	Biopsy + EBRT	5	Seizures	Right occipital, left parietal lobes, vertebral	WBRT	8
Traserra et al. (1990) [20]	M, 52	Left hemilarynx SCC	TL + left ND	1	Left III, IV, and VI nerve palsy, bilateral V hypesthesia	Bilateral CS	-	2
	M, 49	Epiglottis and vallecula SCC	TL, glossectomy, right ND	9	Diplopia and right facial hypesthesia	Right CS	EBRT	3
	M, 43	Left pyriform sinus SCC	TL, bilateral ND	0.5	Headache, diplopia, retroocular pain	Left CS	EBRT	2
Weiss et al. (1994) [2]	F, 64	Laryngeal SCC	TL + PP + bilateral ND + EBRT	9	Diplopia and left VI nerve palsy	PG + CS	Surgery + EBRT	19
de Bree et al. (2001) [1]	F, 53	Supraglottic laryngeal SCC	TL + bilateral ND + EBRT5	7	Diplopia and III palsy	Left CS	Surgery + CT	14
Hardee and Hutchison (2001) [4]	F, 56	Oral SCC	Right hemiglossectomy + ND + EBRT	12	Weakness, vomiting, cerebellar signs	Left cerebellar	Surgery	3
	M, 53	Right tonsil and left soft palate SCC	Left soft palate and right tonsil resection + bilateral ND	0	Hyponatremia	PG	EBRT	8
Uzal et al. (2001) [21]	M, 55	Laryngeal SCC	PL + left ND	9	Diplopia, blurred vision, diabetes insipidus	PG	Surgery + EBRT	6
Dimri et al. (2003) [7]	F, 40	True vocal cords SCC	TL + EBRT	96	Headache, seizures	Bilateral multiple brain	EBRT	3
Sullivan and Smee (2006) [14]	M, 51	Right lower lip SCC	Local excision of the lip	7	Right V1, V2, V3 hypesthesia	Right CS	EBRT	3
González et al. (2007) [23]	M, 63	Left oropharyngeal SCC	Biopsy + EBRT + left ND	7	Diplopia	Bilateral CS	EBRT + CT	6 (still alive)
Al-Khudari et al. (2014) [19]	M, 61	Right palatine tonsil SCC	CT + EBRT	0	Left hemiparesis, blurry vision	Calvarium + bilateral frontoparietal	Surgery + EBRT	6 (still alive)
Leimert et al. (2013) [5]	M, 53	Oral SCC	Radical surgical resection + ND + EBRT	26	Seizures	Right parietal lobe	Surgery	10
Ghosh-Laskar et al. (2016) [11]	M, 57	Base of tongue SCC	EBRT + CT	31	NA	NA	WBRT	3
	M, 69	Soft palate SCC	EBRT + CT	22	NA	NA	WBRT	2
	M, 45	Pyriform sinus SCC	Neoadjuvant CT + surgery + EBRT + CT	8	NA	NA	WBRT	2
	M, 32	Lower alveolus SCC	Surgery + RT + CT	2	NA	NA	WBRT	0.5
	M, 55	Lower alveolus SCC	Surgery + RT + CT	1	NA	NA	WBRT	2
	F, 45	Lower alveolus SCC	EBRT	NA	NA	NA	WBRT	0.5
	M, 50	Lower alveolus SCC	EBRT	NA	NA	NA	WBRT	3
	M, 42	Supraglottic SCC	CT + EBRT	67	NA	NA	WBRT	0.5
	M, 71	Glottis SCC	CT + EBRT	56	NA	NA	WBRT	3
	M, 57	Supraglottic SCC	CT + EBRT	18	NA	NA	WBRT	6
	M, 57	Oral cave SCC	CT + EBRT	2	NA	NA	WBRT	0.5
	M, 46	Tonsil SCC	CT + EBRT	31	NA	NA	WBRT	4
	M, 58	Pyriform sinus SCC	Neoadjuvant CT + surgery + CT + EBRT	15	NA	NA	WBRT	4
	M, 71	Supraglottic SCC	EBRT	2	NA	NA	WBRT	3
	M, 56	Pyriform sinus SCC	CT + EBRT	26	NA	NA	WBRT	2
	F, 65	Oral cave SCC	Surgery + CT + EBRT	6	NA	NA	WBRT	1
	M, 35	Tongue SCC	Surgery + CT + RT	6	NA	NA	WBRT	2
Naruse et al. (2017) [15]	M, 60	Left lower alveolus SCC	Surgery + left ND + CT	4	Speech impairment	Frontobasal bilateral	Surgery	2
Forner et al. (2018) [24]	M, 62	Tongue SCC	Biopsy + CT + EBRT	7	Binocular vertical diplopia	Bilateral CS	Biopsy + CT	NA
Montano et al. (2018) [3]	M, 65	Laryngeal SCC	TL + bilateral ND	36	Right hemiparesis	Left occipital lobe	Surgery + EBRT + CT	7 (still alive)
Grandhe et al. (2019) [25]	M, 42	Hypo and oropharynx, larynx SCC	Surgery + ND + CT + EBRT	6	Headache	Right temporo-occipital lobe	Radiosurgery	NA
Leòn-Ruiz et al. (2019) [26]	M, 71	Supraglottic exophytic lesion SCC	TL + bilateral ND + CT	6	Bilateral tonic-clonic seizures	Frontal osteolytic and left frontoparietal	Surgery + EBRT + CT	5
Cristaudo et al. (2020) [8]	M, 52	Pyriform sinus SCC	CT + EBRT	6	None	Left occipital lobe	Surgery + WBRT	2
Jadhav et al. (2022) [27]	F, 53	Oral cave SCC	None	0	Left VI and VII nerve palsy	Bilateral CS	CT + WBRT	2
Pattanaik et al. (2022) [16]	M, 50	Retromolar trigone SCC	CT + EBRT	1	Low back pain and paraparesis	Multiple leptomeningeal medullar C2-C7	None	0
Present case	M, 64	Laryngeal SCC	PL + bilateral ND	84	Headache, Jacksonian march	Right insular and parietal lobe	Surgery + CT + WBRT	7

The review indicated that the median age of patients with non-nasopharyngeal HNSCC brain metastases was 55 years, with a notable male predominance of 80%. A significant correlation was identified between severe tobacco smoking or alcohol consumption and the development of CNS metastases, present in 88% of the cases. This finding reinforces the well-established link between these lifestyle factors and the etiopathogenesis of HNSCC.

Histologically, moderately differentiated HNSCC emerged as the most common subtype, followed by poorly differentiated variants. Notably, a substantial proportion of primary lesions (28%) were associated with HPV, suggesting a potential viral contribution to the metastatic process [14]. The variety of cranial areas affected by metastases was significant, particularly the cavernous sinuses and pituitary glands, which may be attributed to anatomical contiguity [3,15-17].

The involvement of the cavernous sinuses and pituitary glands in a considerable percentage of cases suggests a potential pattern of spread that warrants further investigation [2,10]. Hypotheses regarding anatomical contiguity, venous drainage anatomy, and histologic aggressiveness offer promising avenues for future research to enhance our understanding of the pathophysiology of CNS metastases in HNSCC [19].

CNS metastases arising from primary HNSCC are rare but represent a devastating progression of the disease. The management of such cases is complicated by the absence of standardized treatment protocols and the typically poor prognosis. Our case contributes to the limited but expanding body of evidence that may inform future clinical practice and guide research efforts aimed at improving outcomes for patients facing this challenging condition.

## Conclusions

This case highlights the complexities involved in managing CNS metastases in HNSCC and underscores the limited survival benefits of aggressive interventions. Given the patient’s neurological decline despite surgical resection, a conservative approach aimed at maintaining quality of life may often be more appropriate. This scenario reinforces the necessity for early recognition of CNS involvement and emphasizes the critical importance of individualized treatment strategies, particularly considering the rarity of these metastases and the absence of standardized protocols.

The review of existing literature further emphasizes the challenges in managing CNS metastases from HNSCC, which are generally associated with a poor prognosis. However, the limited number of reported cases and the variability in treatment approaches prevent definitive conclusions regarding the optimal level of treatment aggressiveness. While some studies indicate that less aggressive management may be suitable for certain patients, additional research is needed to identify specific patient and disease factors that could guide these treatment decisions. Future studies, ideally involving larger and more diverse patient cohorts, should concentrate on identifying predictive molecular markers and developing targeted therapies aimed at improving patient outcomes while minimizing neurocognitive impairment. Comprehensive evaluations of various therapeutic modalities will be essential for refining treatment guidelines and enhancing care for patients with this challenging condition.
